# High-level integration of murine intestinal transcriptomics data highlights the importance of the complement system in mucosal homeostasis

**DOI:** 10.1186/s12864-019-6390-x

**Published:** 2019-12-30

**Authors:** Nirupama Benis, Jerry M. Wells, Mari A. Smits, Soumya Kanti Kar, Bart van der Hee, Vitor A. P. Martins dos Santos, Maria Suarez-Diez, Dirkjan Schokker

**Affiliations:** 1 Host Microbe Interactomics, Wageningen University & Research, Wageningen, The Netherlands; 2 Systems and Synthetic Biology, Wageningen University & Research, Wageningen, The Netherlands; 3 Wageningen Livestock Research, Wageningen University & Research, Wageningen, The Netherlands; 4 Wageningen Bioveterinary Research, Wageningen University, Wageningen, The Netherlands; 5 LifeGlimmer GmbH, Berlin, Germany

**Keywords:** Pathway analysis, Transcriptomics, Data integration, Intestine, Complement pathway, Homeostasis

## Abstract

**Background:**

The mammalian intestine is a complex biological system that exhibits functional plasticity in its response to diverse stimuli to maintain homeostasis. To improve our understanding of this plasticity, we performed a high-level data integration of 14 whole-genome transcriptomics datasets from samples of intestinal mouse mucosa. We used the tool Centrality based Pathway Analysis (CePa), along with information from the Reactome database.

**Results:**

The results show an integrated response of the mouse intestinal mucosa to challenges with agents introduced orally that were expected to perturb homeostasis. We observed that a common set of pathways respond to different stimuli, of which the most reactive was the Regulation of Complement Cascade pathway. Altered expression of the Regulation of Complement Cascade pathway was verified in mouse organoids challenged with different stimuli in vitro*.*

**Conclusions:**

Results of the integrated transcriptomics analysis and data driven experiment suggest an important role of epithelial production of complement and host complement defence factors in the maintenance of homeostasis.

## Background

The mammalian gastrointestinal (GI) tract is crucial for the digestion and absorption of nutrients, energy metabolism, and homeostasis of the gut barrier and mucosal immunity. A number of specialized adaptations of the mammalian mucosal immune system have evolved to maintain a peaceful co-existence with the microbial symbionts while responding appropriately to prevent infection by enteric pathogens [1, 2]. Changes in external factors like the diet or intake of medication can influence microbiota ecology but also host metabolic processes [3–15]. The intestinal epithelium plays an important role in orchestrating innate defences [16, 17] and signalling to the numerous cells of the immune system located underneath the epithelial layer [18–20].

The gut functionalities described above are attributed to groups of genes organised into various functional pathways [21–24] responding to physiological changes. These pathways can be modulated by enteric infection, toxic compounds in food or produced by the microbiota, ionic and osmotic changes as well as substantial variations in nutrient availability. We hypothesized that several pathways are involved in maintaining homeostasis, the regulation of which depends on the type of perturbation. The diverse range of changing conditions encountered at the intestine would require a high-level of functional plasticity compared to other tissues. This theory is supported by the fact that a higher number of genes are specifically expressed in the gut mucosa than that in the heart, liver, kidney, and other organs that carry out a narrower range of functions [25–27]. Transcriptional responses of the intestinal mucosa to individual stimuli or perturbations have been documented widely in literature [15, 28, 29]. However, little is known about which key biological pathways provide functional plasticity in the intestinal mucosa. Interest in understanding this plasticity stems from the current trend to develop (dietary) interventions to optimise gut health and reduce the risk of disease.

Therefore, it is essential to investigate the functional plasticity of mucosal tissues at the functional genomic level in terms of pathways. Such an approach may aid to identify key sets of biosynthetic and signalling pathways involved in the mucosal responses, but also to identify the commonalities and differences in the expression of pathways responding to various environmental and physiological perturbations. To investigate this, we used publicly available gene expression datasets generated from mouse intestinal tissues exposed to orally administered challenges. From the results of the analysis on these datasets we identified the pathway “Regulation of Complement Cascade” that appears to play an important role in the functional plasticity of the intestinal epithelial response to different nutritional, microbial, and chemical challenges.

The complement system consists of several inactive pre-proteins produced in the liver that circulate in the blood which are crucial for efficient clearance of invading organisms. It is part of the innate immune system and activation of the complement cascade plays a key role in the opsonisation of micro-organisms to increase phagocytosis by macrophages and neutrophils at the sites of infection [30]. To avoid complement injury to autologous tissues, complement activation is controlled by a number of fluid-phase and cell surface proteins. Given the importance of this pathway in the GI system and in our high-level data integration we validated the results of our analysis with an in vitro experiment on mouse intestinal organoids. This experimental validation allowed us to, i) validate our data driven experimental design approach, ii) fulfil our aim of gaining more understanding of the functional plasticity of the GI tract.

## Results

### Classification of intestinal gene expression datasets

We identified 14 publicly available datasets meeting the search criteria. We classified them into three broad stimulation categories: Diet (7 experiments); Drug (3 experiments); and Immune Challenge (4 experiments) based on the type of intervention. Stimulations that were given as part of the feed of the animal were classified as ‘Diet’. Stimulation with a substance that is used as medication was classified into the ‘Drug’ category. Any substance that elicits a strong immune response was classified as an ‘Immune Challenge’. It could be argued that the DSS challenge belongs to the Drug category rather than Immune Challenge category as it can be used as a drug. However, since the effects of DSS likely result from a primary epithelial damage leading to the translocation of bacterial antigens, here we have chosen to classify it as an Immune Challenge.

**Fig. 1:**
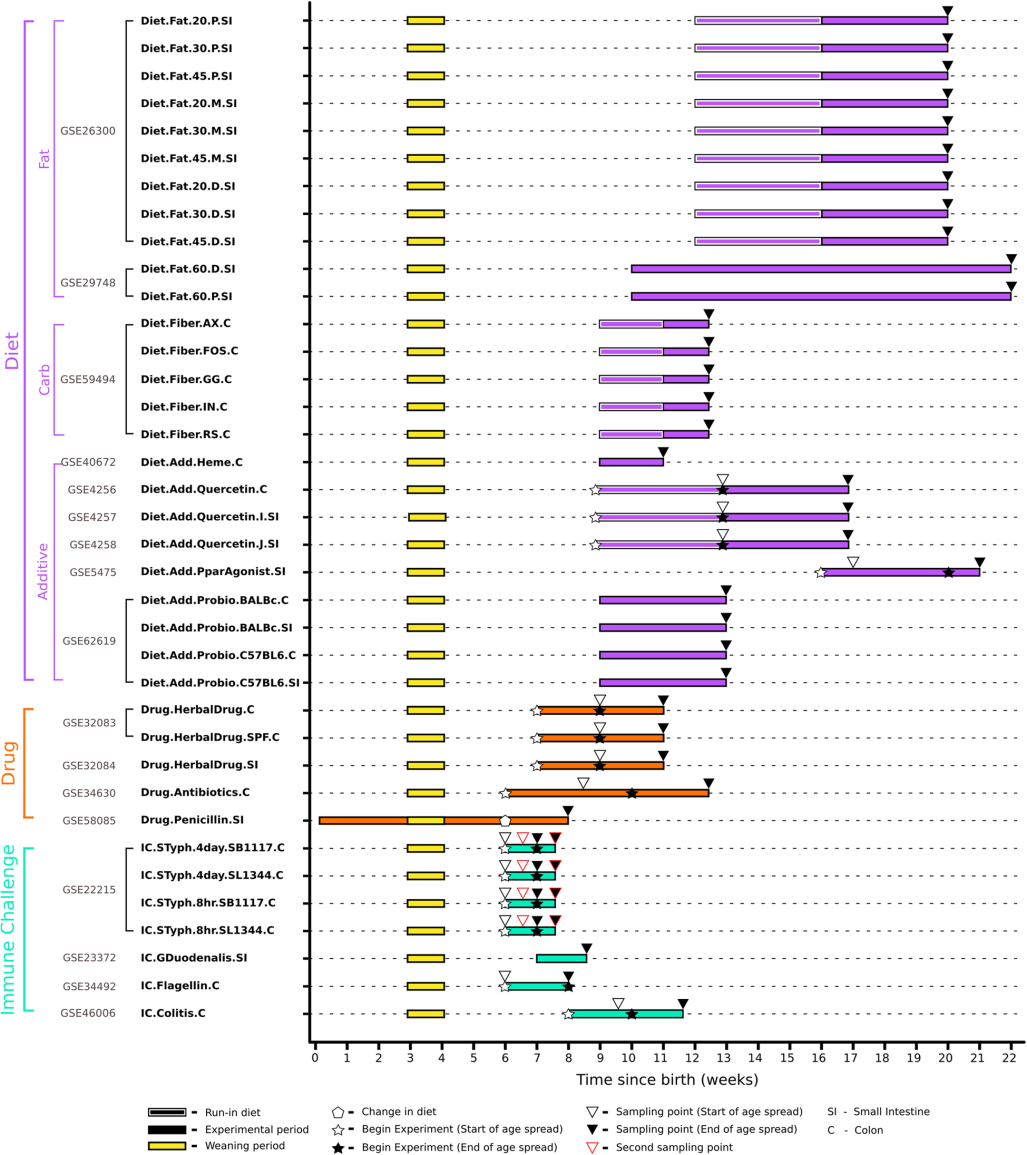
Experimental datasets: The 37 conditions from 14 experiments (with 17 GEO accession numbers) used in this study are detailed in a timeline based on the age of the mice. Mice are selected to be part of an experiment based on weight, hence their age can vary within a range. The stars denote the start of the intervention, an empty star indicates the range of age when the intervention starts, when the age is not the same for all the animals in the group. Triangles denote the end of the interventions, an empty triangle indicates the start of the range of age of the animals. Challenges have been divided in three categories (colour coded): Diet, Drug and Immune Challenge. The names given for each dataset are abbreviated to show the challenge category in the first part of the name, the tissue sampled at the end (SI: small intestine; C: colon) and the text in the middle indicates the nature of challenge. Additional detailed explanations for the abbreviated condition names and the control conditions are given in Additional file 1: Table S1

These 14 experiments comprised of 37 experimental conditions (Additional file 1: Table S1), where a condition is defined as a unique combination of an inbred mouse strain, a specific intervention and an intestinal tissue sampled at a certain time point, as depicted in Fig. 1.

### Significant pathway results for all the datasets

In order to identify pathways specifically regulated by the challenges we used a modified version of the Centrality based Pathway Analysis (CePa) algorithm [31] and the ‘in-reach’ and ‘out-reach’ centrality options. Each analysis was performed on a single comparison, where a comparison is made between a stimulated condition versus the corresponding control in that experiment. We used the Reactome database [32] for the pathway information, which is arranged heirarchically from ‘root’ pathways (very broad), to more specific, ‘leaf’ pathways. We only work with the leaf pathways for the pathway analysis.

For all 37 conditions analysed, 710 pathways were significantly enriched, see Additional file 2: Table S2 for *p*-values of these pathways. The majority of these pathways (84%) were significant in both centrality measures whereas about 11% were significant only in the ‘in-reach’ centrality and 5% significant in only the ‘out-reach’ centrality. An overview of the pathway analysis results is given in Additional file 4: Figure S1. This figure shows that the responses are partially influenced by the tissue that was sampled since the red points (large intestine) mostly separate from the green points (small intestine). The number of signifcantly regulated pathways for each condition is highly variable, with the maximum being 377 from the experiment with Heme (Diet.Add.Heme.C) and the minimum being 37 in the experiment where 20% of the energy in the diet came from fat (Diet.Fat.20.P.SI). The average number of signifcantly regulated pathways is highest in the conditions belonging to the Diet category with 132 pathways, the second highest is Immune Challenge with 101 pathways followed by Drug with 87 pathways on average.

### Comparison of the significant pathways in the three experimental challenge categories

**Fig. 2:**
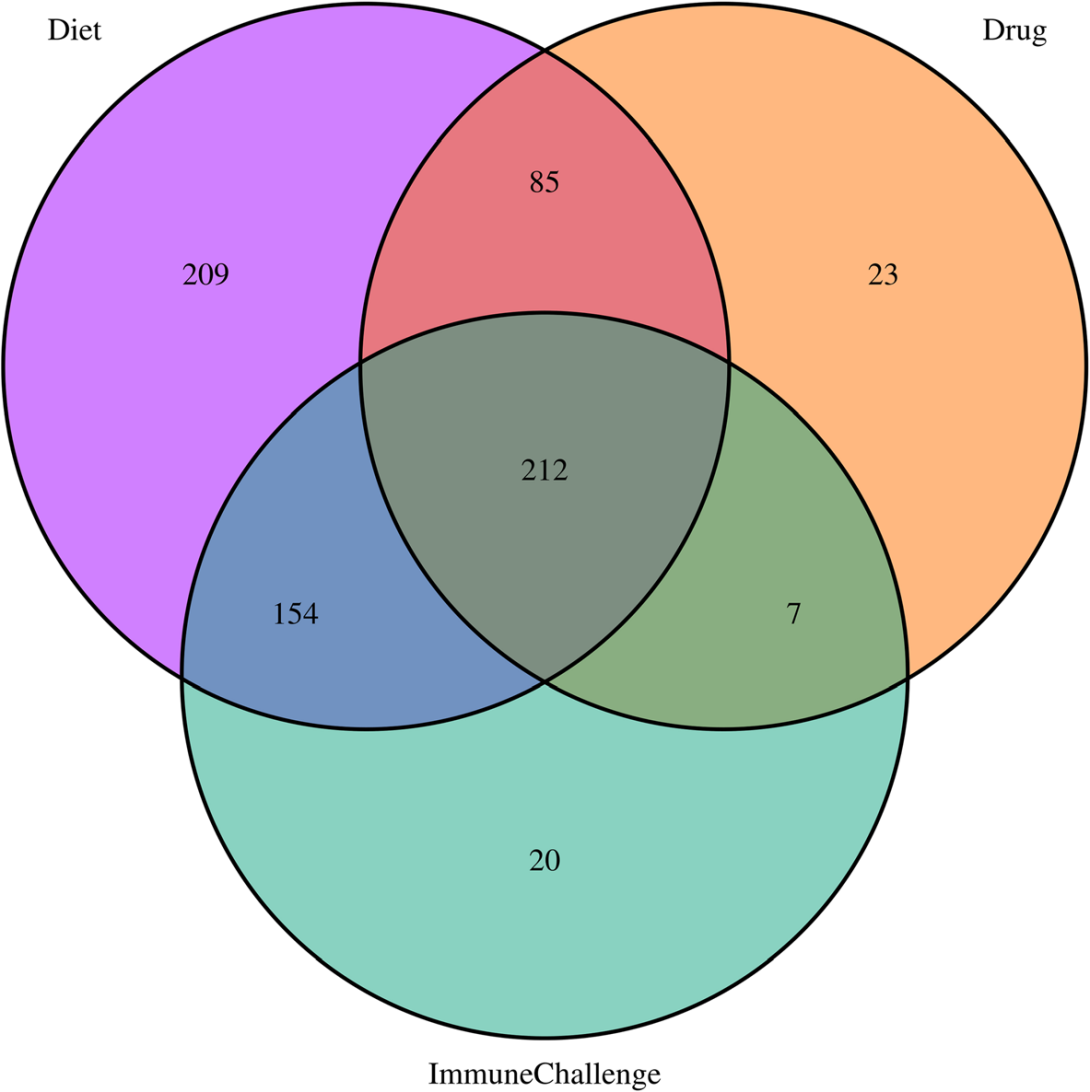
Number of leaf pathways enriched in differentially expressed genes in the three challenge categories. The three circles are indicative of the significant leaf pathways in the datasets belonging to one of the three challenge categories Diet, Drug, or Immune Challenge. The 212 common pathways of all the three classes are indicated in the centre

There was a large overlap in the results between the three categories (Fig. 2). In addition, there were several pathways unique to each challenge category, the largest number of pathways were identified in the Diet category, which also had the largest number of experimental conditions in our study. There is a large number (212) of pathways that are shared among all challenge categories (Fig. 2). These 212 pathways belong to 24 of the 27 root pathways. The distribution of the 212 leaf pathways among the 24 root pathways of Reactome is shown in Table 1. The roots with the largest number of results are Metabolism, Disease, and Signalling pathways, but the proportion of common leaf pathways is similar to the proportion of all the leaf pathways in the roots in the Reactome database.

**Table 1:** The 24 root pathways common to the three challenge categories. The names of the root pathways are given in the first column. The second column shows the number of leaf pathways in a particular root and this number is shown as a percentage of all the leaf pathways in the root pathway in the database

Root pathways	Common Leaf pathways	Ratio of common leaf pathways in the root pathway
Metabolism	54	22%
Disease	53	24%
Signalling Pathways	47	32%
Immune System	29	33%
Cell Cycle	20	30%
Gene Expression	13	18%
Hemostasis	11	44%
Programmed Cell Death	7	26%
Transcription	6	29%
Metabolism of proteins	4	8%
Chromatin organization	3	60%
Circadian Clock	3	100%
Developmental Biology	2	5%
DNA Replication	2	18%
Extracellular matrix organization	2	18%
Membrane Trafficking	2	18%
Transmembrane transport of small molecules	2	6%
Binding and Uptake of Ligands by Scavenger Receptors	1	20%
Cell-Cell communication	1	13%
Cellular responses to stress	1	9%
DNA Repair	1	4%
Muscle contraction	1	50%
Organelle biogenesis and maintenance	1	9%
Post-Elongation Processing of the Transcript	1	25%

### Regulation of pathways shared by the challenge categories

In order to investigate the most differently regulated pathways among the three conditions, the 212 common pathways were ranked based on a Difference Score. The Difference Score was calculated based on the mean node scores of the pathway nodes. The node scores of the pathway are a t-statistic which is a differential value between the experimental condition and the control. A mean of the node scores in the pathway under experimental conditions within one challenge category was calculated to end up with three scores for a pathway. The sum of the difference between the three mean values, the Difference Score was used to rank the list of common pathways. Table 2 shows the top 10 pathways ranked by this method and their corresponding Difference Scores.

**Table 2:** Top 10 pathways with the most difference in expression between the three perturbation classes. The second column shows the specific experimental conditions in the perturbation classes that were most different. The Difference Score was calculated using the t-values of the pathway nodes in the given conditions

Pathway Name	Most different conditions	Difference Score
Regulation of Complement cascade	Diet.Add.Probio.BALBc.C - Drug.HerbalDrug.SPF.C - IC.STyph.4 day.SB1117.C	21.07
APC/C:Cdc20 mediated degradation of Cyclin B	Diet.Add.Heme.C - Drug.Antibiotics.C - IC.GDuodenalis.SI	20.07
Cdc20:Phospho-APC/C mediated degradation of Cyclin A	Diet.Add.Heme.C - Drug.Antibiotics.C - IC.Colitis.C	17.92
Inactivation of APC/C via direct inhibition of the APC/C complex	Diet.Add.Heme.C - Drug.Antibiotics.C - IC.Colitis.C	17.42
Formation of the HIV-1 Early Elongation Complex	Diet.Add.Probio.C57BL6.C - Drug.Antibiotics.C - IC.STyph.4 day.SL1344.C	16.96
Formation of HIV elongation complex in the absence of HIV Tat	Diet.Add.Probio.C57BL6.C - Drug.Antibiotics.C - IC.STyph.4 day.SL1344.C	16.09
Amplification of signal from unattached kinetochores via a MAD2 inhibitory signal	Diet.Add.Heme.C - Drug.HerbalDrug.C - IC.Colitis.C	16.07
Signal regulatory protein (SIRP) family interactions	Diet.Fat.30.M.SI - Drug.Penicillin.SI - IC.STyph.4 day.SB1117.C	15.87
Termination of O-glycan biosynthesis	Diet.Add.Heme.C - Drug.HerbalDrug.C - IC.STyph.4 day.SB1117.C	14.73
Formation of HIV-1 elongation complex containing HIV-1 Tat	Diet.Add.Probio.C57BL6.C - Drug.Antibiotics.C - IC.STyph.4 day.SL1344.C	13.87

**Fig. 3:**
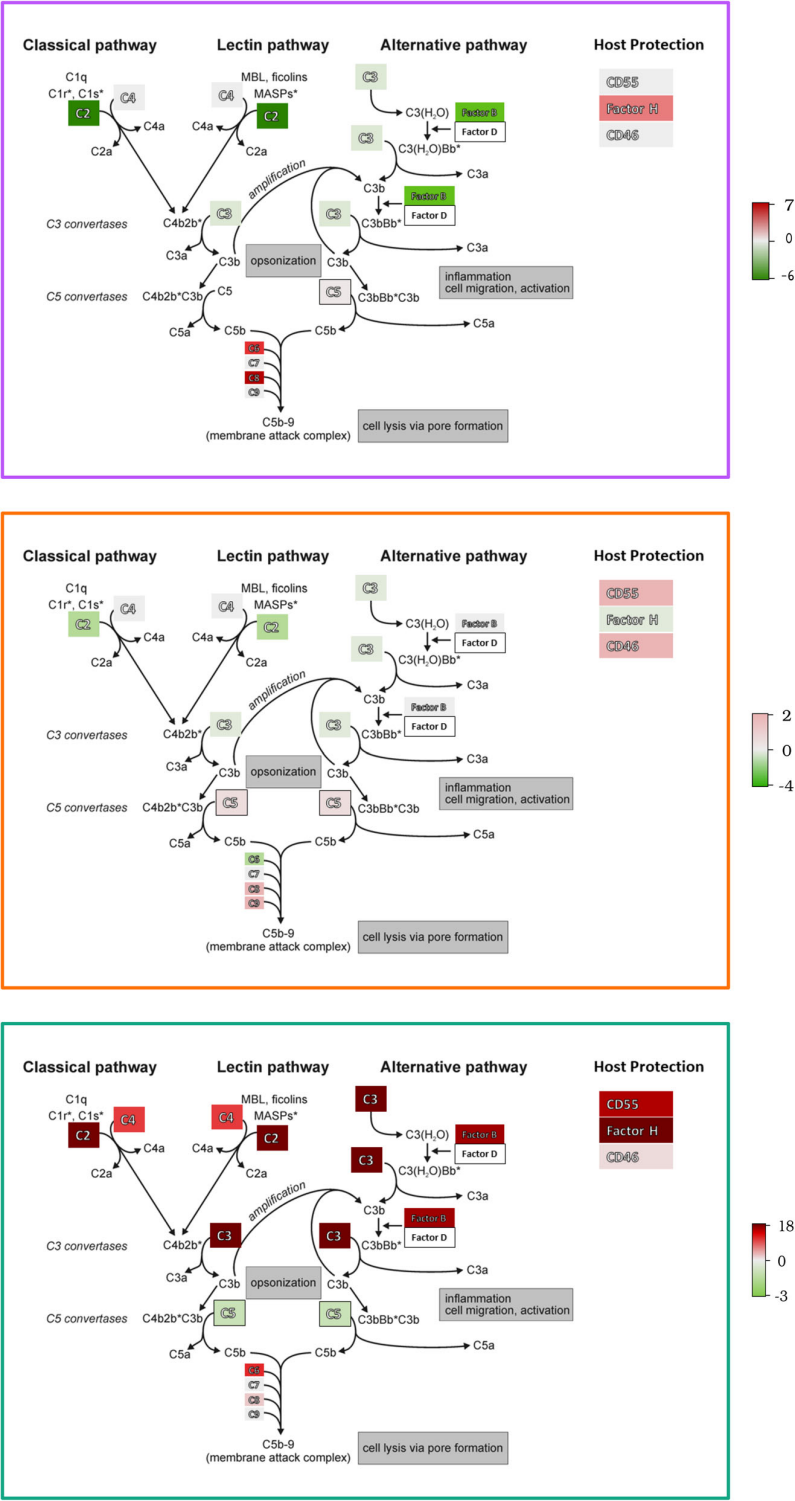
The three complement pathways leading to enhanced phagocytosis of microorganisms. The binding of C3b to a receptor expressed on the surface of phagocytes and formation of the C5 convertase which generates chemotactic factors C5a and C3a, and the membrane attack complex for lysis of microbial membranes are depicted in the figure. Names of enzymatic products or complexes are shown. Common gene names are shown in boxes and are shaded in red when positively regulated in the datasets included in this study and green when negatively regulated. Each panel shows the complement pathway; however, it is superimposed with the pathway gene expression measurements for three different experimental conditions, one from each challenge category. Panel **a** represents the expression of the genes in the condition Diet.Fat.45.P.SI, panel **b**, Drug.Penicillin.SI and panel **c**, IC.STyph.4 day.SL1344.C. The legend shows the range of differential regulation. Complement-mediated defence mechanisms are shown in grey boxes. Modified from Microbes Online by Srijana Khanal [33]

The largest differences between the three challenge categories and gene expression of nodes were found in the innate immunity pathway, ‘Regulation of Complement Cascade’ (Fig. 3). All the genes in the Reactome pathway ‘Regulation of Complement Cascade’, with their differential expression values, are provided in Additional file 3: Table S3. The Regulation of Complement Cascade pathway was significantly regulated in 17 conditions in vivo (11 Diet, 3 of 7 Drug and 3 out of 5 Immune Challenge conditions). In the Diet conditions containing added fat, the complement factor genes mentioned above were also increased in expression (Fig. 3, Additional file 3: Table S3). In the Drug category, penicillin (Drug.Penicillin.SI), expression of the complement pathway genes highlighted in Fig. 3 were unaffected or mostly down-regulated whereas some were upregulated by the herbal drug conditions (Drug.HerbalDrug.SI, Drug.HerbalDrug.SPF.C). The biggest change was in the Immune Challenge category infection, where *Salmonella* upregulated the complement factors C4, C2, C3. These complement factors are required for activation of the complement cascade via the classical pathway and mannose lectin pathway (Fig. 3). C3 and factor B which are required for activation of the alternative pathway were also upregulated by some conditions in the Immune Challenge category (Fig. 3). Although C5 expression was only moderately upregulated by some of the Immune Challenge conditions, C6 was strongly upregulated. The other complement factors forming the membrane attack complex (MAC) on the surface of microorganisms were not strongly regulated under any of the conditions (Additional file 3: Table S3). The host protection factors (Fig. 3) CD55, CD46 and factor H, which are important for protection of host cell membranes when complement activation is triggered by microbes, displayed similar expression patterns as the complement factors (Fig. 3, Additional file 3: Table S3).

### Response of representative stimulants of the three challenge categories in mice intestinal organoids

To investigate the regulation of genes in the pathway ‘Regulation of Complement Cascade’ under similar conditions as tested in vivo, we performed experiments on adult stem cell derived ileal organoids from mouse. The organoids contained all the main epithelial cell lineages found in the tissue of origin, including comprehensive components of the complement cascade [34–36].

As defined stimuli, we used TNFα, an inflammatory cytokine-induced by infection or activation of inflammatory pathways, bacterial flagellin, an agonist of an innate immune receptor Toll Like Receptor 5 (TLR5) and a pharmacological agonist of PPARα a transcription factor and a major regulator of lipid metabolism.

**Fig. 4:**
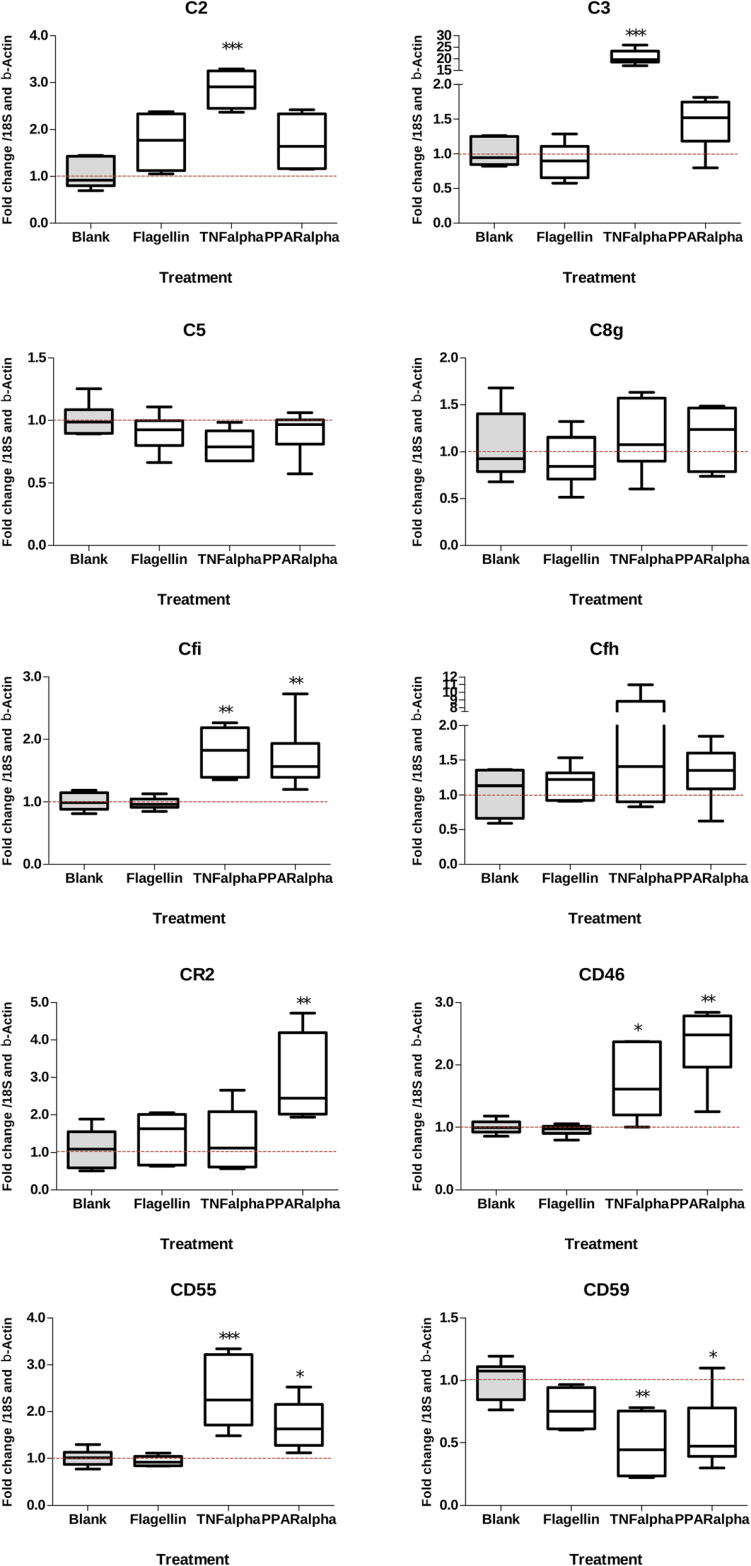
Expression of 10 chosen genes from the ‘Regulation of Complement Cascade’ pathways with significance calculated with ANOVA. Each graph contains information on different genes, the x-axis contains information on the treatment of the organoids and the y-axis has the fold change of the control genes. Data were analysed using Prism statistical software (v5.0, Graphpad, San Diego, US), measured for normality using the Kolmogorov-Smirnov test, and represented as Box and Whisker plots. A t-statistic test was performed on the RT-qPCR results of the 10 genes using the same methods as on the nodes in the gene set pathway analysis of CePa. All data were considered significantly different from the Blank (indicated in grey) when *P* < alpha (0.05) and indicated with * (*P* < 0.05 = *, *P* < 0.01 = **, *P* < 0.001 = ***)

The inflammatory cytokine TNFα which is induced by infection (e.g. with pathogenic *Salmonella*) induces expression of all complement related genes except *C5, C8GH* and *CR2* (Fig. 4). In contrast, none of the genes were significantly altered in expression by incubation with flagellin, despite its ability to activate TLR5 signalling on HEK reporter cells expression TLR (data not shown). None of the genes related to the Regulation of Complement Cascade pathway were altered by the PPARα agonist. Instead, we observed the PPARα agonist significantly altered the expression of host receptors and CFI which are involved in protection of the host from complement activation.

## Discussion

By integrating the results of experiments in which intestinal homeostasis was perturbed by completely different challenges, including probiotics, antibiotics, infectious agents, and major dietary components, we were able to investigate the plasticity of the GI tissue in terms of engaging various (biological) pathways. To the best of our knowledge, this type of study, has not yet been performed on this scale, focusing on one tissue and different types of challenges. By grouping the different challenge conditions in categories comprising drugs, dietary ingredients or potentially inflammatory agents, we grouped the responses of the mucosal tissue to facilitate broader comparisons. The results revealed pathways which are regulated in all categories. In addition, we observed large differences in the expression profiles of pathway genes between different exposure conditions, in some cases, irrespective of the challenge category. By focussing on the commonly regulated pathways, we show that the gut mucosa employs similar pathway systems. However, these pathway systems are used in different combinations and with different intra-pathway gene expression profiles, to respond to different exposures.

### High-level data integration and pathway level analysis

The most important criteria for dataset selection was the age at which the mice were sampled, because the mucosal immune system and intestinal microbiota of mammals is known to change dramatically around weaning [37]. This holds true for mice [38, 39], therefore we only included datasets where the mice were sampled two or more weeks after weaning. The inclusion criteria provided datasets that are comparable, but they still differed in many aspects such as the use of microarray platforms, in sampling of the tissue and in the control conditions. By using controls within an experiment, we expect to only eliminate differences caused by platforms and retain biological influences like the type of tissue and the perturbation. Therefore, we used a high-level data integration method that started with the identification of differently expressed pathways as detected within an individual experiment and/or experimental condition. Before the pathway analysis the discriminating factor between the datasets was the platform in which the data was measured (data not shown). After the high-level data integration, the main differences among the datasets is the sampled tissue and the type of perturbation, see Additional file 4: Figure S1.

We used the CePa algorithm that considers a pathway’s topology by using different network centrality measures. Based on the biological information behind the pathways, we decided to use two centrality calculations, ‘In-reach’ and ‘Out-reach’ to capture regulation of pathways down-stream (important for signalling pathways) and up-stream (for metabolic pathways) respectively.

Most of the results were significant in both the centrality calculations irrespective of the type of pathway. This apparent indifference to topology is also observed by Bayerlova et al. [40] in a different pathway database using a variety of algorithms. In the aforementioned study, among the algorithms that used pathway topology, the CePa GSA algorithm performed consistently well. But, as also discussed in Khatri et al. [41], in order to make the most use of pathway topology it is important to be able to better annotate the edges between pathway nodes.

### Several pathways were regulated by all three challenge categories

The results of the integrated pathway analysis show a notable overlap in the pathway response between the three challenge categories. The Diet category has the highest number of leaf pathway results, and also the highest number of experimental conditions. Surprisingly, most of the results from Drug and Immune Challenges were also shared by the Diet class. The results demonstrate that there is a group of pathways that are commonly regulated by interventions which perturb homeostasis. These common pathways contribute to a major extent towards the capability of the intestinal mucosa to display a high-level of functional plasticity. Most of the transcriptomics data used in this study came from intestinal scrapings which is greatly enriched for different types of epithelial cells involved in innate immunity and cross-talk with the immune cells in the lamina propria [2]. The different functions of these epithelial cells contribute to the functional plasticity of the epithelium. The results of this study revealed another layer of plasticity which is based on the specific use of a common set of pathways. These common pathways are significantly differentially expressed compared to controls in at least one experimental condition in each category. Simplifying the regulatory output of a whole pathway is difficult due to issues like up-regulation of the expression of inhibitory molecules.

### The pathway regulation of complement Cascade is regulated by multiple intestinal challenges

One of the 212 common pathways which responds differently to the challenge categories is the ‘Regulation of Complement Cascade’. This pathway showed the largest difference in node expression profiles between the three challenge categories as shown in Table 2. The local production of complement factors must be important in intestinal homeostasis as the pathway shows maximum difference between the challenge categories and is regulated in several experimental conditions. Although the complement cascade is mentioned in two of the experiments used in this study [42, 43], the effects of the experimental conditions on the complement cascade were not explored in detail.

Complement factors involved in complement activation by one of three pathways are produced in the liver and enter the circulation. Complement factors reach tissue sites of infection through acute inflammation which results in the exudation of fluid and plasma proteins and an emigration of leukocytes into the extravascular compartment. Our observation that key complement factors involved in the complement activation pathways (e.g. C2, C3, C4, factor B), complement control and host protection (CD55, CD46, CFI, CFH), are increased in expression by epithelial cells exposed to infectious challenge, suggests that local complement production may be needed in the intestine as an early defence mechanism against encounters with microorganisms due to a dysfunctional barrier or infection. This idea has been proposed before [44, 45] especially with regards to inflammatory conditions and here we have explored this hypothesis in our dataset. This hypothesis was supported by our finding that transcription of complement system genes was regulated in mouse organoids in response to agonists of different signalling pathways. Only complement factors involved in the early stages of pathway activation and opsonization were strongly upregulated under inflammatory challenge conditions. Activation of the complement pathways in the mucosal tissues would lead to early opsonization of microorganisms and production of chemokines such as C5a to attract immune cells. The observed upregulation of host factors involved in protection of autologous cell membranes from complement damage is also compatible with the hypothesis that complement activation occurs in the mucosal tissue. The upregulation of C2, C3, C4, and factor B but not C5 and the complement factors (C6-C9) required for generation of the membrane attack complex (MAC), which is an important effector protein, is consistent with other literature describing complement factor expression in colorectal carcinoma cell lines stimulated with various cytokines [46, 47]. C3 and C4 transcripts have been localised to intestinal crypts in biopsies from Crohn’s patients [48]. This is consistent with the notion that epithelial cells can be induced to express complement factors needed for opsonisation of invading bacteria, but not cell lysis.

In the Diet category of challenges high fat diets strongly upregulated transcription of complement pathway genes, which may be due to a low-grade inflammation and a hyperpermeable gut [49, 50]. Interestingly, depletion of the microbiota with penicillin reduced pathway expression, suggesting that the microbiota contribute to ‘tonic’ stimulation of the complement related pathways via stimulation of innate immunity.

To confirm that the intestinal epithelium could express complement factors in response to signalling pathways targeted by the dietary challenges, we stimulated small intestinal crypt derived mouse organoids with TNFα, flagellin, an agonist of PPARα, or culture medium as a control and compared the relative transcript abundance of selected complement pathway genes by reverse transcription polymerase chain reaction (RT-PCR). TNFα is known to be induced by invasive infection by enteric infection with Salmonella [51], which was used in the Immune Challenge category [52]. Furthermore, receptors for TNFα are present on intestinal cells and signal in response to TNFα [53]. TLR5 was chosen because flagellin was orally administered in one of the studies in immune challenge category and PPARα agonist was selected because it was administered in one of the studies in the diet category with the aim of altering lipid metabolism [42]. One of the characteristics of inflammation is compromised barrier function, leading to a cascade of events in the lamina propria, e.g. TNFα secretion by invading immune cells, or bacterial fragment translocation [2]. As some TLR receptors may only be active or signal via the basolateral membrane of enterocytes [2, 54] we stimulated intact organoids. Genes to be observed were selected based on biological significance and fluctuation in the datasets that were analysed and RT-qPCR was performed on those mice genes (written in italics to differentiate from human genes). Stimulation with the inflammatory cytokine TNFα increased transcription of complement factors *C2*, *C3* and regulatory proteins *Cfi, Cd46* and *Cd55*, whereas expression of *C5, C8* and *Cfh* was not significantly changed and *Cd59* was significantly down-regulated. The agonist of PPARα significantly up-regulated transcription of regulatory proteins *Cd55, Cr2, Cd46,* and *Cfi* and significantly down-regulated expression of *Cd59*. Surprisingly, flagellin (which was shown to activate TLR5 in an intestinal cancer cell line [55]) did not significantly alter expression of any of these genes. The reasons for this are unclear but may be due to aberrant expression or regulation of TLR in intestinal cancer cells which are known to display biological variations such as aneuploidy, chromosome rearrangements or mutations [56]. It has also been proposed that TLR5 signalling is tightly controlled in epithelial cells to avoid chronic inflammatory responses to bacterial MAMPs from the intestinal lumen, and that expression of this receptor is exclusively present on Paneth cells in the small intestine [54]. Having TLR5 exclusively expressed in Paneth cells could explain the low responsiveness to flagellin, since this cell type is abundant at low levels in organoids [57]. Interestingly, activation of the PPARα pathway also increased expression of protective factors *Cd55, Cd46, Cfi* and *Cr2* which allows the complement system to play a role in B cell activation and maturation. This links PPARα to regulation of complement cascade in the gut and the effects of high fat diets on this pathway in vivo*.*

CD55 and CD46 have other functions, besides their role in protection of host cells from complement activation which may be relevant for intestinal homeostasis. CD55 binds to the neutrophil receptor CD97 expressed on neutrophils to promote neutrophil migration through the epithelium [58]. Binding of antibodies to CD46 on Caco-2 cells was shown to induce intracellular signalling and improved cell proliferation and wound healing [59].

## Conclusions

In conclusion, high-level data integration of transcriptomics datasets from intestinal tissue from in vivo experiments was a valuable approach to identify common pathways associated with functional plasticity and intestinal homeostasis. The identified pathways are regulated in different combinations to generate different physiological responses, or genes within a pathway are differentially regulated contributing further to the plasticity. The “Regulation of Complement Cascade” pathway is one of many pathways regulated by multiple intestinal challenges suggesting it is an important mechanism in the periphery of the intestine, which might have poor access to circulating complement components from the blood. Epithelial expression of complement factors involved in opsonisation and chemotaxis of host phagocytes, but not formation of the MAC complex, indicates a primary function in opsonisation of microbes and chemotaxis of host immune cells. Coincident with the increased intestinal expression of complement factors is the expression of host factors involved in complement control and protection such as CD55 and CD46 which have secondary functions in innate immunity and wound healing.

## Methods

### Datasets

The R tool *GEOmetadb* [60] was used to search Gene Expression Omnibus (GEO) [57, 61] for publicly available datasets generated from intestinal samples of mice. We selected 14 transcriptomics experiments (17 GEO datasets) from the 450 available (as of 07-07-2015) which analysed any intestinal tissue section from an intervention in weaned mice. All selected experiments used single channel microarrays with at least 3 biological replicates and were published on GEO between 2006 and 2014. Three of the datasets were obtained using Illumina microarray platforms whereas the others were obtained using versions of the Affymetrix platform. Most experimental data were obtained from inbred C57BL6J mice, but there were two experiments that used BALB/c mice, one that used the 129S1/svlmj mouse strain and another that used IQI mice. Eleven experiments were performed on female mice and three experiments on male mice.

There were two experiments where the fat content of the diet was increased by reducing the carbohydrate portion of the diet [11, 62]. The former experiment provided three different levels of fat (20, 30 and 45% of total energy in the diet) and performed transcriptomics on three sections of the small intestine. The latter experiment provided 60% fat as total energy in the diet and measured gene expression in RNA isolated from two halves of the small intestine. One experiment tested five different fibres which were substituted for part of the corn starch in the diet and the transcriptomics data was generated from the colon [63]. The rest of the dietary interventions were additives or supplements to the mouse diet. In one experiment, dietary heme was added to a high fat diet and the response was measured in the colon [64]. In another experiment quercetin was added to the standard diet and the response was measured in two parts of the small intestine (jejunum and ileum), as well as in the colon [65]. Another experiment added a synthetic PPARα agonist to the diet and a microarray analysis was performed on the small intestine [42]. One experiment tested a probiotic on two strains of mice and two sections of the intestine (small and large intestine) [66]. There are three experiments where a drug was administered to the animals. In one of these experiments a herbal drug was tested on wild-type and specific pathogen free mice, and the response measured in the small intestine and colon [43]. The other 2 drug experiments involved administration of antibiotics. One study utilised a mix of several antibiotics to strongly deplete the abundance of gut bacteria, and investigated gene expression in the colon [67]. In the other experiment with antibiotics, a low dose of penicillin was administered daily to the animals from an early age and the diet was also changed before measuring the response in the small intestine [68]. There were 2 challenge experiments, one with *Salmonella Typhimurium* [52], where the colon was sampled andanother involved the parasite *Giardia duodenalis* [69] where the small intestine was sampled. The effect of flagellin from *Salmonella enterica* serovar Typhimurium [70] on the intestinal immune system and the severity of DSS-induced colitis was investigated [71] in the colon. More details on the experimental conditions and the control conditions can also be found in Additional file 1: Table S1.

### Data pre-processing

Using *GEOQuery* [72], we downloaded normalised datasets of preselected experiments from GEO. In each experiment, the normalization was performed with one of the following methods, GCRMA, RMA, MAS5 or quantile normalization. Our high-level integration approach does not require uniform normalization, so in each case we preferred the method chosen by the authors of the original study. The probes were mapped to mouse Entrez identifiers using the annotation files from the platform that was used for microarray analysis. After a quality check using Principle Component Analysis plots, these mice gene identifiers were then mapped to their human homologs using the NCBI HomoloGene database [RRID:SCR_002924]. Code snippets for these steps can be found on the GitHub page https://github.com/nirupamaBenis/PathwayLevelDataIntegration.

### Pathway database

The analysis of all the datasets was performed using pathways from the Reactome database [RRID:SCR_003485], a freely accessible and a manually curated database available in different formats. Pathways from Reactome were downloaded in the BioPAX [RRID:SCR_009881] (Biological Pathway Exchange) [73] format (version 51) from the official website. These pathways were then converted to a pathway catalogue object in R that can be used by the pathway analysis algorithm. This was accomplished by using the pathway2Graph function from the R package *rBiopaxParser* [RRID:SCR_002744] [74, 75].

All pathways in the Reactome database are arranged in a hierarchy, larger ‘root’ pathways consist of more and more specific pathways, ending in several ‘leaf’ pathways. This hierarchy is depicted in a simplified cartoon in the inset of Additional file 5: Figure S2. The main image in Additional file 5: Figure S2 shows a network of all the root pathways in Reactome version 51. These 27 root pathways contain 1639 pathways within their hierarchy, of which 950 are leaf pathways.

### Pathway analysis

We used a modified version of the algorithm CePa (Centrality based Pathway analysis), which uses pathways as networks where the nodes in a pathway could be small molecules (compounds), macro-molecules (proteins or RNA) or complexes (more than one protein). The topological information of the pathway is used to assign weights to each node using centralities. The user can choose between one of four centrality measures, in-degree (number of edges that are directed towards the node), out-degree (number of edges that are directed outwards from the node), in-reach (longest path that brings information to the node) and out-reach (longest path that directs out of the node), with another option of giving equal weight to all nodes. This centrality information is used along with the expression data to give a list of significantly enriched pathways for given conditions vs their controls. There are two methods of using the expression data in the CePa package, Over-Representation Analysis (ORA) and Gene Set Analysis (GSA). ORA usually takes a list of differentially expressed genes which could be ranked with *p*-values or fold changes. GSA takes the entire matrix of expression values to find enriched pathways and this is the method we chose to use.

The gene expression data is mapped to the nodes of the pathways, when the node is a protein the expression value of the corresponding gene is used as such. When the node is a complex, the largest component from a Principle Component Analysis of the expression values of all the corresponding proteins is assigned as the node expression value. Subsequently, these expression values are inputted in a t-statistic to obtain a differential expression value for each node, which can be positive or negative based on the up- or down-regulation of that protein. This differential node value is multiplied with the centrality-based weight of the node to obtain a final node value. This calculation is performed for each of the nodes in the pathway and all these values are averaged to obtain a pathway level score. The pathway level score is then tested for significance by substituting random expression values in the same pathway calculations and comparing the obtained value with the original dataset and the value obtained with the randomized data. The fraction of the iterations on which a higher score is obtained with the randomized data is used to represent the p-value. This p-value calculation was modified from the original CePa function which randomizes the replicates of the tested conditions. As we work with a minimum of three samples per condition, we modified this calculation to be able to handle smaller sample sizes. The original algorithm randomized the data across samples in order to calculate the significance of a pathway score. We decided to randomize the expression values by genes so that there is a larger chance of the values being truly random and thus without a biological signal. Because the hierarchical nature of the pathway database implies dependence between the pathways, we decided not to perform a multiple testing correction.

We weighted the nodes with the in-reach and out-reach centrality calculations, because they assign higher weights to the nodes down-stream and up-stream of the pathway respectively. In this way, we can capture signalling pathways, where the effectors are more likely to be down-stream of the pathway. However, we did not rule out the metabolic pathways where the enzymes are generally up-stream in a pathway. The threshold of the *p*-values was set at 0.01 to compensate for the lack of multiple testing correction.

### Intestinal organoid cultures

Three dimensional (3-D) crypt derived murine intestinal organoids were grown as described in literature [34, 76–78]. Briefly, a 2 cm duodenal section was opened longitudinally and washed in ice-cold phosphate-buffered saline solution (PBS). After scraping excess villi, the tissue was transferred to PBS containing 2.5 mM EDTA and incubated for 30 min. Following incubation, the sections were washed with PBS and remaining residue was passed on a 70 μm cell strainer, pelleted at 300 x g for 5 min, and suspended in matrigel basement membrane (Growth factor reduced, Corning) at a density of 50–100 crypts per 50 μl. After inversed polymerization at 37 °C for > 10 min, 600 μl basal culture medium (DMEM/F12) was added, enriched with mouse EGF, Hepes 1 M (Invitrogen), N-acetylcysteine (Sigma), B-27 (Thermo-Fisher), Noggin, and R-spondin. The culture was passaged 1:4 every 7 days by mechanical disruption and re-suspension in fresh Matrigel. All experiments were performed after 2 passages of the organoid cultures.

### Stimulation of organoids and reverse transcriptase-quantitative PCR

The 3-D organoids were stimulated with TNFα (10 ng/ml), a PPARα agonist (WY14643 0.1% v/v), and flagellin (200 ng/ml) for 6 h before total RNA was extracted with the Qiagen mini-kit according to manufacturer’s instructions along with a 15 min DNAse step. Purity and integrity measurements were performed on a DS-11 spectrophotometer (DeNovix) and 1 μg total RNA was reverse transcribed into cDNA using a QScript kit (Quantabio). Quantification of gene expression (RT-qPCR) was performed using a Rotor-gene Q2 plex RT-cycler (Qiagen) on primers specified in Table 3 with the rotor-gene SYBR green PCR kit, also from Qiagen. These genes were selected based on their representative contribution to the pathway ‘Regulation of Complement Cascade’. Relative expression levels were calculated following methods described in [81] using individual amplification values, with 18S and β-Actin as endogenous control genes for normalization.

**Table 3:** Primers used for RT-qPCR. The information on the primers used to quantify 10 genes is given in each row along with the publication from which this sequence was obtained

Gene	Forward	Reverse	AT (°C)	Amplicon (bp)	Ref
*C2*	CTCATCCGCGTTTACTCCAT	TGTTCTGTTCGATGCTCAGG	60	178	[79]
*C3*	AGCAGGTCATCAAGTCAGGC	GATGTAGCTGGTGTTGGGCT	60	167	[79]
*C5*	AGGGTACTTTGCCTGCTGAA	TGTGAAGGTGCTCTTGGATG	60	173	[79]
*Cfh*	CGTGAATGTGGTGCAGATGGG	AGAATTTCCACACATCGTGGCT	60	248	[79]
*Cfi*	TTCCACTGGGTGTTCGTGAC	TAAAGGCACACTCCGCCAAA	60	126	[79]
*Cd46*	CCAGGGCCAGATAAGTTTTC	TATTTCGCCAGCTCCTGATA	60	153	[79]
*Cd55*	CTCTGTTGCTGCTGTCCC	CGAATAATATGCCGGTTG	60	477	[80]
*Cd59*	TAAGTGAGTTCCTGGCAACC	AGGGCCTGTGAAGATTATGA	60	152	[79]
*Cr2*	CCTGCTCCTCTCTGTAAACT	GATCTGACTGCTTCCACTCA	60	162	[79]
*C8g*	CTGGCTCCTTGTGGCTGT	CGAAACTCTGGTAGTCGGTCTC	60	257	Author

Untreated 3-D organoids were used as control to obtain relative gene expression values of the 10 chosen complement pathway genes when stimulated by the three treatments. Genes encoding C3 and C5 were selected as they are key factors in the three complement activation pathways. Genes encoding CD46, CD55 and CD59 were chosen because they are involved in protection of host membranes when complement pathway is activated. The other five genes (*C8GH, CFI, CFH, CR1,* and *C2*) were chosen because their expression varied substantially under the 17 experimental conditions where the ‘Regulation of Complement Cascade’ pathway was significantly affected. Complement Factor H and Factor I are involved in the regulation of complement activation, C2 is a component required for activation of the classical and alternate pathways. The murine *CR2* contains 25 exons; a common first exon is spliced to exon 2 and to exon 9 in transcripts encoding CR1 and CR2 which encode receptors binding complement complexes on host immune cells.

## Supplementary information

**Additional file 1: Table S1.** Description of the 37 conditions. This table describes the abbreviated names of experimental conditions as used in the paper and the control conditions in each of the experiments.

**Additional file 2: Table S2.** Significant pathways with p-values in each experimental condition both centralities. This table has the p-values of all the significant leaf pathways in the 37 experimental conditions in both the in-reach and out-reach centrality calculations. If the p-value of the pathway was above the threshold of 0.01 an empty space is shown in the table. The p-values of at least one of the centrality calculations have to be lower than the threshold.

**Additional file 3: Table S3.** Differential expression of genes in “Regulation of Complement Cascade” pathway. This table contains the differential gene expression values of 24 genes in 17 experimental conditions. The differential values were obtained with a T test of the experimental condition vs the control in that experiment.

**Additional file 4: Figure S1.** PCA of all the significant pathways over the experimental conditions. Green points represent p-values from the pathway analysis from the small intestine and the red ones from the large intestine. Circles represent experimental conditions from the Diet category, the triangles are from the Drug category and squares from the Immune Challenge category.

**Additional file 5: Figure S2.** Network of Reactome root pathway. The nodes in this network represent the 27 root pathways as present in Reactome v51 and the edges indicate the ‘leaf’ pathways shared by connected root pathways. The thickness of the edges indicates the number of leaf pathways shared by the nodes. The nodes are labelled with the names of the root pathways and the number of enclosed leaf pathways is given between brackets. The inset shows a simplified example of root and leaf pathways, where the cartoon has one root pathway with three leaf pathways.

## Data Availability

All the datasets used in the analysis were retrieved from the online repository Gene Expression Omnibus and the identifiers for each dataset is provided in the manuscript as are the hyperlinks to publications based on the data. R code and detailed instructions to perform these analyses can be found at https://github.com/nirupamaBenis/PathwayLevelDataIntegration.

## Abbreviations

BioPAX: Biological Pathway eXchange
CePa: CEntrality based Pathway Analysis
DMEM: Dulbecco’s Modified Eagle Medium
DSS: Dextran Sulfate Sodium
EDTA: Ethylenediaminetetraacetic acid
EGF: Epidermal Growth Factor
GCRMA: Guanine Cytosine Robust Multi-array Analysis
GEO: Gene Expression Omnibus
GI: Gastrointestinal tract
GSA: Gene Set Analysis
MAC: Membrane Attack Complex
ORA: Over-Representation Analysis
PBS: Phosphate-Buffered Saline
RMA: Robust Multi-array Averaging
RT-PCR: Reverse Transcription Polymerase Chain Reaction
