# Supplementary figures and images for: High-level integration of murine intestinal transcriptomics data highlights the importance of the complement system in mucosal homeostasis
Source: BMC Genomics. 2019 Dec 30;20:1028. doi: 10.1186/s12864-019-6390-x (PMC6937694; doi:10.1186/s12864-019-6390-x)

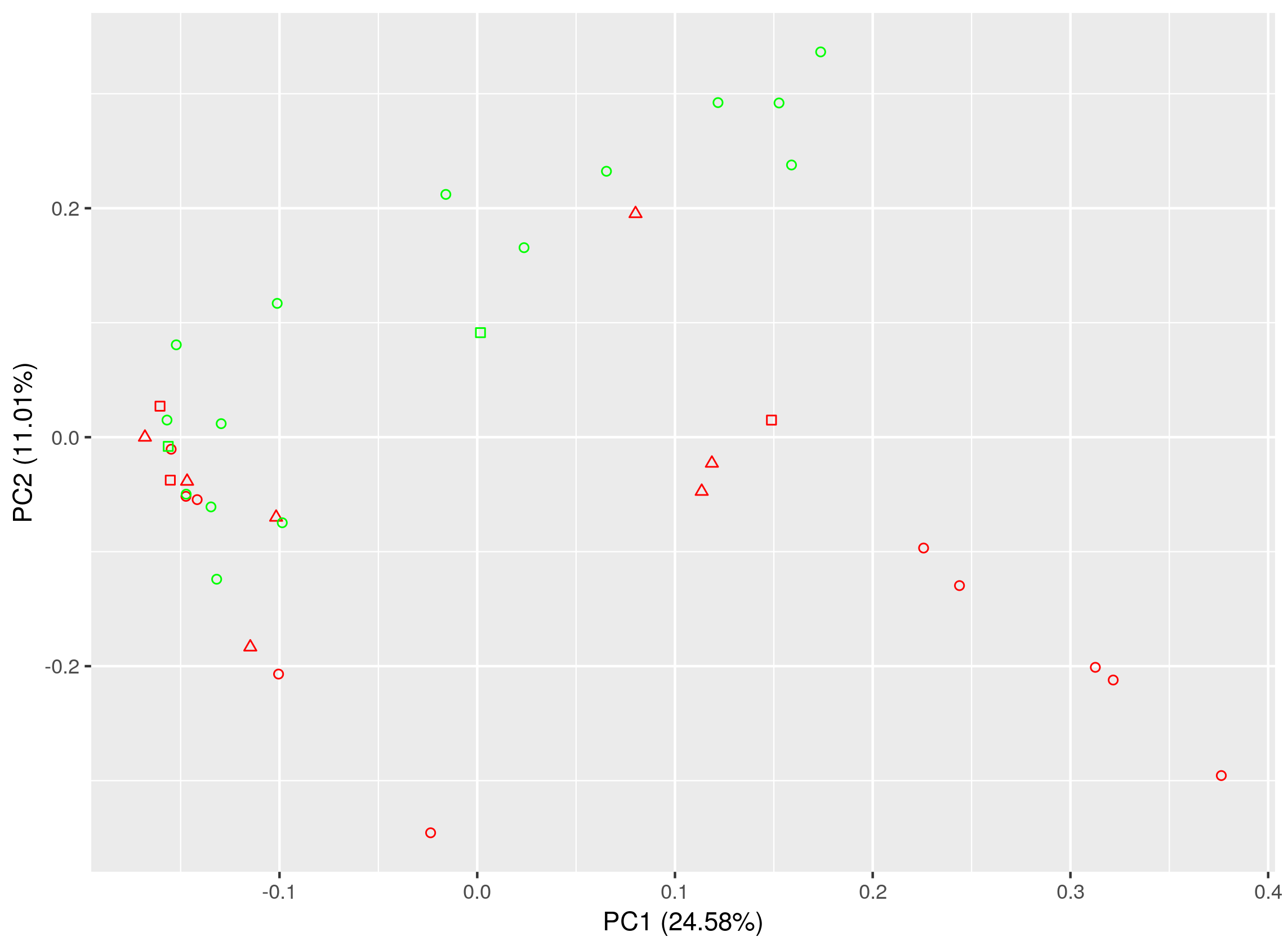

Supplement: Supplementary file 4 — Additional file 4: Figure S1. PCA of all the significant pathways over the experimental conditions. Green points represent p-values from the pathway analysis from the small intestine and the red ones from the large intestine. Circles represent experimental conditions from the Diet category, the triangles are from the Drug category and squares from the Immune Challenge category. [file 12864_2019_6390_MOESM4_ESM.png]

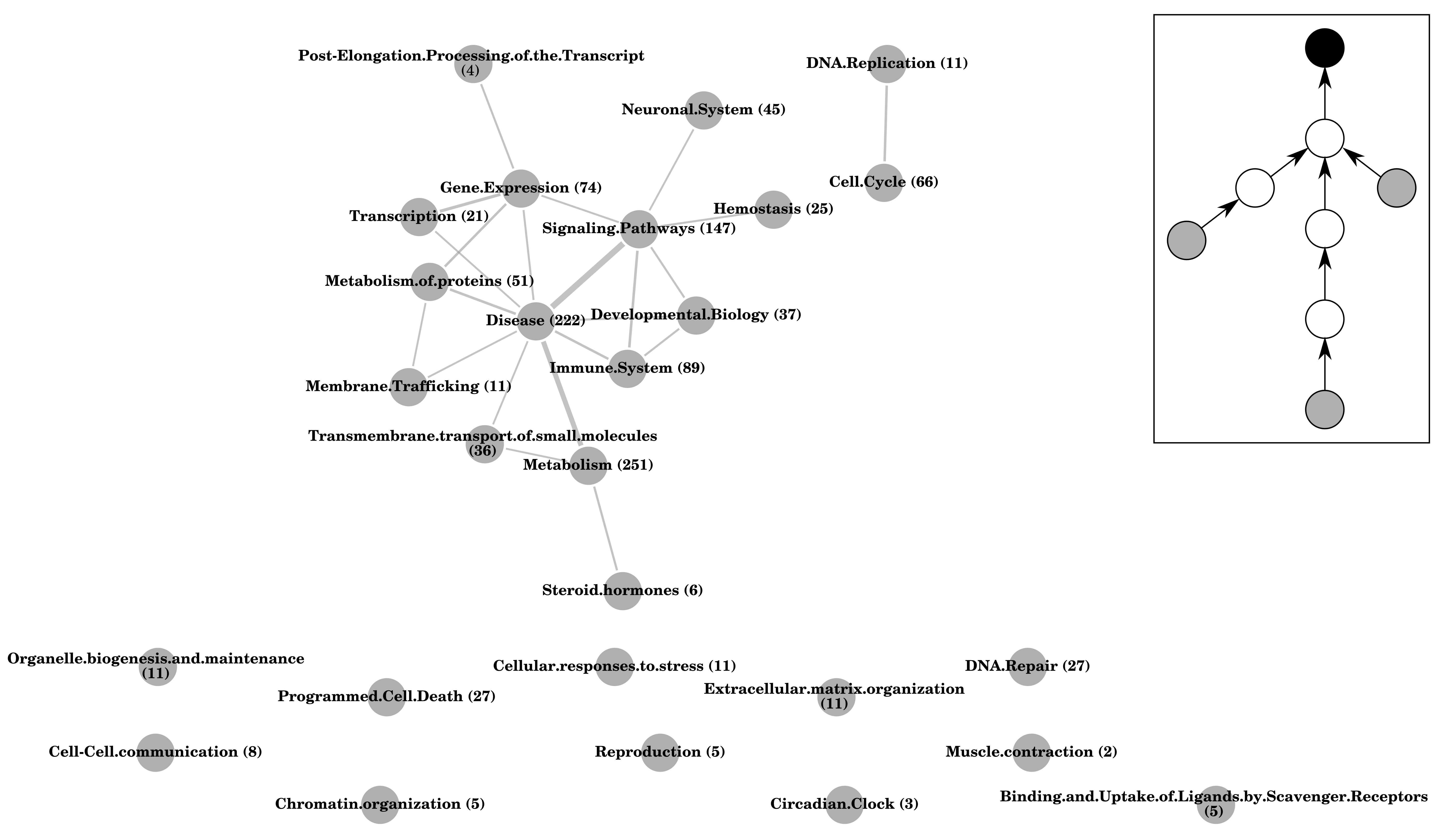

Supplement: Supplementary file 5 — Additional file 5: Figure S2. Network of Reactome root pathway. The nodes in this network represent the 27 root pathways as present in Reactome v51 and the edges indicate the ‘leaf’ pathways shared by connected root pathways. The thickness of the edges indicates the number of leaf pathways shared by the nodes. The nodes are labelled with the names of the root pathways and the number of enclosed leaf pathways is given between brackets. The inset shows a simplified example of root and leaf pathways, where the cartoon has one root pathway with three leaf pathways. [file 12864_2019_6390_MOESM5_ESM.png]
